# Exploring expected and perceived facilitators and barriers of an indicated prevention strategy to prevent future long-term sickness absence; a qualitative study among employers and employees

**DOI:** 10.1186/s12889-021-10322-w

**Published:** 2021-02-04

**Authors:** Sophie H. Klasen, Ludovic G. P. M. van Amelsvoort, Inge Houkes, Nicole W. H. Jansen, IJmert Kant

**Affiliations:** 1 Department of Epidemiology, Faculty of Health, Medicine and Life Sciences, CAPHRI School for Public Health and Primary Care, Maastricht University, P.O. Box 616, 6200 MD Maastricht, The Netherlands; 2 Department of Social Medicine, Faculty of Health, Medicine and Life Sciences, CAPHRI School for Public Health and Primary Care, Maastricht University, Maastricht, The Netherlands

**Keywords:** Preventive health services, Sick leave, Needs assessment, Qualitative research

## Abstract

**Background:**

An indicated prevention strategy (IPS), consisting of a screening questionnaire and early treatment, was found to be effective for the prevention of future long-term sickness absence (LTSA) in two large Dutch RCT’s. This IPS aims to detect employees who have a high risk to become absent, and subsequently offer them early treatment. Despite the overall effectiveness, only a few companies have implemented this strategy so far. This suggests that companies may not be convinced of the (cost) effectiveness of this strategy yet. In companies where IPS has been implemented, screenings uptake and adherence to early treatment appeared to be moderate, indicating that both employees and employers might perceive barriers.

**Methods:**

The aim of this qualitative study was to explore the expected and perceived facilitators and barriers for the implementation of the IPS. Semi-structured interviews were conducted with 9 employers and 11 employees (acquainted and unacquainted with IPS) from large companies. Purposive sampling was used to recruit participants. All interviews were transcribed and analyzed thematically.

**Results:**

The employers believed they were primarily responsible for psychological and work-related health complaints and SA, while the employees felt responsible for health complaints related to their lifestyle. According to the employees, the responsibility of the employer was solely related to work-related health. This finding exposed a relation with the health culture, which was solely based on creating a safe work environment, omitting psychological health issues. The efficacy of this IPS regarding reducing SA was estimated positive, however, the efficacy regarding LTSA was questioned. Fear of a privacy breach was often mentioned by the respondents as an important barrier.

**Conclusions:**

This study showed that the health culture within a company may be important for the perceived responsibility towards SA and health. A health culture which primarily focuses on physical complaints may raise barriers for the adoption and implementation of this preventive strategy. Participant’ perceptions of the nature of LTSA and the fact that not all participants were familiar with the exact content and phasing of IPS may have doubted the efficacy regarding LTSA. This study provides important clues for future and improved implementation of IPS.

**Supplementary Information:**

The online version contains supplementary material available at 10.1186/s12889-021-10322-w.

## Background

Long-term sickness absence (LTSA) is a major occupational and public health issue in Western countries [1]. In the Netherlands, employers generally are obliged to pay at least 70% of the employee’s salary in the first 2 years of sickness absence (SA) (regardless the cause), often resulting in high costs for employers [2–4]. Returning to work after a period of LTSA is often difficult and may even lead to permanent work disability, affecting employee wellbeing and resulting in further costs for employers and society [5–8]. SA is a complex, multi-factorial phenomenon, which often has personal, work-related, and social determinants [9, 10]. For the prevention of SA, return to work strategies to prevent SA, as well interventions based on principles of universal or selective prevention (which only focus on one or two explaining factors) appear to be less effective [8, 11, 12]. Because often due to the multifactorial etiology of SA, a more comprehensive approach is needed [11, 12].

An indicated prevention strategy might be more promising in the prevention of LTSA since it focuses on multiple factors concerning SA. Indicated prevention strategies are designed to prevent the onset of a disease or health issues, the individual is not yet sick but shows early warning signs and is, therefore ‘at high-risk’ [13]. Innovative indicated prevention strategies have been developed, which showed their efficacy in predicting and preventing future SA [12, 14, 15]. This study will focus on one of these strategies, henceforward called ‘IPS’. The IPS consists of a screening questionnaire, followed by early consultation with the occupational health professional (OHP) for employees at high risk for future LTSA. Following the consultation, early treatment starts with the OHP or another health professional [16]. This IPS has been evaluated in two large Dutch RCTs and appears to be effective concerning the prediction and prevention of future SA and improving the mental health status of employees [12, 14, 16, 17].

Although the potential benefits of these strategies are high, their application/implementation is still rather low [5]. This suggests that employers/companies are not convinced of the (cost) effectiveness of this IPS yet. Moreover, in the companies where IPS has been implemented, screenings uptake and participation in the early intervention is only moderate, suggesting that employees may encounter barriers or hindering factors with respect to participation in the IPS [18]. Many different stakeholders with different interests are involved in workplace health and disability prevention programs, including this IPS. This may complicate the adoption and implementation process of such programs [19–21]. The IPS involves (i) the employer who organizes and bears the cost of screening all of the employees and early interventions for employees identified to be at risk for future SA, (ii) employees who are invited to fill in the screening questionnaire, (iii) employees identified as being at high risk for future SA who are invited for early intervention, and (iv) the OHP who will provide consultation and early intervention. These stakeholders all have different interests regarding the prevention of LTSA, and thus the perceived facilitators and barriers will probably differ, which may inhibit the adoption and implementation of the strategy. Therefore, it is very important to explore the perceived and experienced facilitators and barriers of adoption and implementation of this strategy.

Earlier studies into general, universal, or selective occupational health prevention have investigated relevant facilitators and barriers, which could also apply to this IPS. Improving the productivity and well-being of employees, reducing healthcare costs, and indirect costs related to absenteeism and permanent disability were mentioned as important facilitators by employers [22, 23]. Employees expected improvements in physical as well as psychological health and improvements in their general well-being [22, 24]. Relevant barriers for employers are the high cost of the screening and intervention, logistical issues, and time scarcity [25]. Barriers according to the employees were related to privacy issues and fear of discrimination or stigmatization at work [26]. The early character of this intervention, before employees, actually report sick, and the primary focus on high-risk employees for future LTSA may reveal different facilitators and barriers. A comparable study showed with the use of a survey that questions concerning risk perceptions regarding diabetes, cardiovascular disease and chronic kidney disease created misconceptions. The high-risk classification may create difficulties, while employees may be unaware of the true meaning of being at high-risk [27].

The perspective of the occupational physician (OP) of this IPS has been studied earlier. This study by de Brouwer et al. [28] showed that important barriers from the perspective of OPs were amongst others; communication skills, appropriate training for the OHP and privacy issues related to labeling of high risk employees. The current study will therefore only explore the views from the employers and employees.

It is expected that acquaintance and/or experience with this IPS could also affect the facilitators and barriers, and hence on its adoption and implementation in organizations. Acquaintance with this IPS could resolve the initial concerns related to a preventive intervention or shed light on other important issues which were not foreseen. To our knowledge, no earlier research has investigated whether acquaintance with a preventive strategy will affect how the barriers and facilitators are perceived.

This study aims to explore potential facilitators or barriers regarding this IPS, from the perspectives of employers and employees acquainted/unacquainted with the strategy. We believe that beliefs about these facilitators and barriers are determined by the employers/employee’s perceptions, norms, and values, this study therefore requires a qualitative approach. Our main research question is: *What are the facilitators/barriers of an indicated prevention strategy preventing future LTSA according employers and employees who are acquainted or unacquainted with the IPS?*

## Methods

In order to explore the expected and perceived facilitators and barriers of an indicated prevention strategy (IPS), a qualitative design was chosen. We performed semi-structured interviews with employers and employees. The IPS context under study consisted of a screening phase, using a screening questionnaire and a subsequent intervention in employees identified by the screening phase as being at high risk for future LTSA.

### Screening questionnaire

The screening questionnaire, Balansmeter, includes 34 predictors, each measured by multiple-choice or dichotomous questions. The questions cover different domains: work environment (e.g. working conditions, psychological job demands), characteristics of the private situation, (mental) health status, demographic factors, and SA history. Using an algorithm based on the weighted factors of the individual items of the model, a total score can be calculated, with higher scores indicating a higher risk for future LTSA [14]. A pre-determined cut-off point is used to identify high-risk employees. Different versions have been developed for office and industry workers, with separate algorithms for male and female employees [14]. Screening data generated by this questionnaire are part of an employee’s medical file and as prescribed by Dutch law, may not be shared with the employer, except with the worker’s explicit consent. In the daily practice of the companies that have already implemented the preventive strategy, employees are invited to participate every 3 years. The 3 year frequency has not been validated in earlier studies, however this is in line with the frequency of occupational prevention interventions.

### Early consultation and intervention

**Fig. 1:**
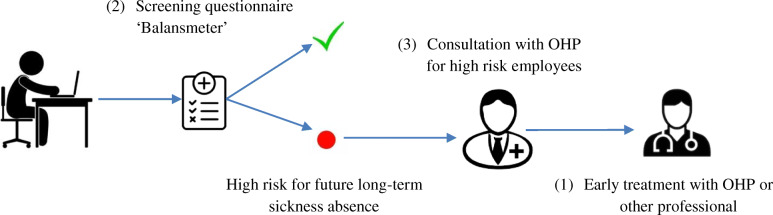
Indicated prevention strategy; prediction and early consultation to prevent future LTSA

Employees at high risk according to the screening questionnaire are invited for an extensive, one-to-one consultation with an occupational health professional (OHP). The OHP was chosen as the expert for consulting with high-risk employees because s/he is specifically trained to recognize work-related and non-work-related conditions and their interactions. The structured early consultation involves several steps, during which the results of the screening questionnaire will be discussed and a broad range of additional anamneses can be performed to consider options for treatment or guidance. A special training is available for the OHPs to facilitate working with the preventive questionnaire, but this training is not obligatory [28]. This consult may then result in a targeted intervention focusing on the specific complaints presented by the employee. The targeted intervention may consist of various conventional treatments, ranging from additional socio-medical counseling by the occupational physician to psychotherapy, counseling by a social worker, or specialized and/or intensified care for a specific disease. A graphic overview of the IPS is shown in Fig. 1.

### Study design, sample and procedure

We employed purposive sampling to recruit employers and employees of varying age (almost all 35–65) and acquainted/not acquainted with the IPS. All employees worked for the same two sites of a petrochemist, this petrochemist has multiple departments were some had implemented this strategy in the past and others had not. The employers were chosen from a university, a Dutch bank, a consultancy company, and a petrochemist (same as the employees). Based on the job levels, we expect the education level of the employees to vary from low to high. The job functions of the employers presume a high educational level.

Employees were considered acquainted with this IPS when they had filled in the screening questionnaire of which some were additionally invited by the OHP for early treatment. Employees were considered unacquainted with this strategy when they had only heard of this strategy and did not fill in the screening questionnaire. The employers were considered to be acquainted with this IPS when they had used the intervention themselves or had a lot of knowledge about this IPS. Employers were seen as unacquainted with this IPS when they knew only basic information about this strategy. Especially the beliefs of the unacquainted employees and employers were considered very important to obtain insights into potential reservations regarding this strategy. For privacy reasons, we could not make a further distinction in acquaintance with this IPS. For the same reason, the researchers did not know whether respondents had been at high or low risk in the past for LTSA. We aimed to include large companies in this study, while this IPS has so far only been implemented in large companies (> 10.000 employees). Investigating the perspectives of similarly large organizations, as the views of employers and employees from Small and Medium-sized Enterprises (SMEs) might be different due to different contexts, structures, cultures and quality of occupational healthcare. Employees and employers were chosen from different large institutions, acquainted and unacquainted with the IPS. An overview of the respondents is shown in Table 1. All respondents were invited for the interview via email, in which the aim of the study was explained.

**Table 1:** Characteristics of the study subjects

	Stakeholder	Gender	Company	Acquaintance with IPS
1	Employer	Female	Bank	Yes
2	Employer	Female	Bank	Yes
3	Employer	Female	Consultancy	Yes
4	Employer	Male	Petrochemist	Yes
5	Employer	Male	University	No
6	Employer	Male	University	No
7	Employer	Male	Petrochemist	No
8	Employer	Male	Petrochemist	No
9	Employer	Female	Petrochemist	No
10	Employee	Male	Petrochemist	yes
11	Employee	Male	Petrochemist	yes
12	Employee	Male	Petrochemist	yes
13	Employee	Male	Petrochemist	yes
14	Employee	Male	Petrochemist	yes
15	Employee	Male	Petrochemist	yes
16	Employee	Male	Petrochemist	yes
17	Employee	Male	Petrochemist	No
18	Employee	Male	Petrochemist	No
19	Employee	Male	Petrochemist	No
20	Employee	Male	Petrochemist	No

### Data collection

All respondents were invited for a face-to-face interview which took place between June 2017 and March 2018. All interviews were conducted in a private room at the respondents’ workplace and were conducted in Dutch by the first author (SK). A topic list was used for each interview (Additional file 1). The topic list gave the first author a starting point, and according to their answers, it was decided how many more questions needed to be asked about a certain topic to get a good idea of the perception. The topic list was used to obtain a more in-depth discussion. The topics were derived from the relevant literature with a focus on the values, barriers, and facilitators of preventive interventions [23, 25, 26]. Core topics, like the expected effect of this IPS on LTSA, the effect on multiple facets of health, and the level of trust in this strategy, were similar for all stakeholders. Stakeholder-specific topics were added, e.g. costs of the strategy for the employers and fear of a privacy breach for the employees, to be able to evaluate more stakeholder-specific views.

Prior to each interview, the respondents were informed by the researcher by e-mail about the purpose of this study followed by a summary of the IPS and its results in the past. Before the start of each interview, the IPS was again explained by the interviewer, to provide the respondents with basic knowledge about the strategy. It was stated that all information given would be treated confidentially. Verbal informed consent was requested before each interview, and the ethical clearance is provided by FHML REC from Maastricht University (FHML-REC/2019/020). The interviews were taped and lasted 40 min on average. They were digitally recorded and transcribed verbatim by an independent assistant. The transcribed interviews were all in Dutch, but the quotes used for this article were translated into English. Data saturation was reached, after approximately seven interviews with the employees and six interviews with the employers, meaning that no new themes or sub-themes emerged from the data.

### Data analysis

The interviews were analyzed thematically, which resulted in main themes with further sub-categorization [29]. This approach enables deductive reasoning while also permitting new themes to emerge from the data. One researcher coded the interviews, and three researchers (IJK, LvA, IH) assessed the coding phase. The coding labels were discussed and adjusted, or coding themes were added with the help of the same researchers who acted as peer reviewers (IJK, LvA, IH). Additional file 2 gives an overview of all the themes with their corresponding codes. Nvivo version 11 was used for the data analysis.

## Results

Three main themes emerged from the data: ‘Values’, ‘Facilitators’, and ‘Side effects and barriers’, each with its sub-themes. To give a clear overview, we categorized the main themes and sub-themes in Table 2. It appeared that the interviewees’ ideas about responsibility for SA and health and the presence of a health culture were important underlying values of perceived barriers and facilitators of the IPS. For the facilitators, we focused specifically on the benefits this strategy could provide for SA, future LTSA, and health.

**Table 2:** Main themes and sub-themes items for employers and employees

Main themes	Sub-themes
Values	**Responsibility**
	• Perceived responsibility for SA
	• Perceived responsibility for health
	**Health culture**
	• Presence of a health culture
Facilitators	**Expected and perceived positive effects**
	• Effect of IPS on future SA
	• Effect of IPS on future LTSA
	• Effect of IPS on health
Side effects & barriers	**Expected and perceived side effects employer**
	• Costs (for the IPS)
	• ROI
	• Costs and benefit balance
	• Healthcare costs
	• Healthcare use
	**Expected and perceived side effects employee**
	• Costs (own payment for preventive care)
	• Restraint own payment for use of preventive care
	• Attitude towards employer if he pays for preventive care
	• Healthcare use
	**Adoption barriers**
	• Trust in the privacy of information
	• Confidence in the IPS
	• Discrimination/stigmatization
	**Practical implementation barriers**
	• Insufficient time to fill out the questionnaire
	• Communication issues with the IPS
	• Low -frequency of the IPS

We distinguished between the expected and perceived side effects for employers and employees. The expected/perceived side effects were related to costs for both the employer and the employee, but in a different manner and concerning the effects on healthcare use. A wide variety of barriers was mentioned which were further divided in adoption barriers and barriers which could obstruct the implementation of the IPS. Adoption barriers were often related to privacy issues, confidence in the strategy, and fear of discrimination. The implementation barriers were more pragmatic, like insufficient time to fill out the questionnaire, communication issues with the IPS, and the low-frequency of the IPS. The narrative is organized according to this division of main themes and sub-themes.

### Values

#### Perceived responsibility for SA

All employers independent of acquaintance/unacquaintance perceived the reduction of SA as a major responsibility of theirs and many agreed it was also the responsibility of the employee. Employers did not feel responsible for issues such as the flu, but mainly for work-related or psychological issues. [Employer8] *“I do not mean flu or something like that, but really when it comes to psychological complaints, then I feel clear responsibility.”* One unacquainted employer mentioned feeling almost 100% responsible for SA related to psychological issues.

All employees regardless their level of acquaintance with the IPS, felt there was a shared responsibility for SA with their employer. [Employee18] *“Yes, if it has to do with my own lifestyle I do not think so, but if it has to do with the absenteeism due to the nature of work or the substances that are present at the worksite, then I think it’s the employer’s responsibility.”* The employer was held responsible if SA was related to work, the work environment, or work-related stress. The employees felt it was their responsibility if SA was a result of their lifestyle.

#### Perceived responsibility for health

Most of the employers regardless their level of acquaintance with the IPS, believed that health is a shared responsibility with their employees. Often, they felt responsible when the health complaints were work-related, like stress or pressure from work tasks. [Employer7] *“I am responsible that the employee can do his work well and we also have a role in the long-term effects of stress and work pressure.”* If bad health was a result of lifestyle choices, they did not feel this responsibility, and only a few felt the need to interfere with lifestyle choices, when the latter negatively affected the employee’s work.

Many employees regardless their level of acquaintance with the IPS, believed the responsibility for their health was as much their responsibility as their employer’s. According to them, the employer was responsible for creating a safe work environment, and the employees were responsible for having a healthy lifestyle. [Employee20] *“Fifty-fifty; As an employer, you are responsible for creating a safe working environment and ensuring that people feel comfortable in their working environment. The employee has a great responsibility to keep himself healthy and vital to be fit for his purpose.”* In comparison with employees who were unacquainted with the strategy, some employees acquainted with the strategy, thought the employer was less responsible for their health.

#### Health culture

According to all employers acquainted with the strategy, health was embedded in the culture of the organization, with attention being paid to psychological issues, mindfulness sessions, and healthy living workshops and programs. Many employers unacquainted with the strategy mentioned the absence of a health culture. Some mentioned that the health culture was related to safety, which was the primary focus for them. [Employer9] “*Safety is our number one priority, but this is not the case for health, while health actually also belongs to safety.*” For them, a health culture was established by creating a safe place to work where they also focused on sports and healthy eating programs. Here countering psychological issues to improve the health situation of their employees is not part of the health culture.

Almost all employees regardless their level of acquaintance with the IPS, associated a health culture with safety, healthy eating, and sports programs. They often felt the organization did not meet their expectations to improve their health situation. Some employees unacquainted with the strategy mentioned that the health culture was focused on physical health which was related to their work and not on the health complaints outside work. [Employee20] “*Focus is very much on physical health and preventing damage during working hours and less on long-term, not work-related aspects.*” Attention to their psychological complaints did not seem to be embedded in the health culture.

### Facilitators

#### IPS effect on future SA

Some of the employers acquainted with the strategy believed this IPS would decrease SA, however, others thought this decrease would be limited due to only a small amount of high-risk employees. The employers unacquainted with the strategy often believed in the preventive effect of the strategy and the benefit of insight in the current health state. [Employer3] *“So yes, in that sense it is preventive, whether we prevent dropout … I do not know. I find it complicated whether we might find people earlier. It is, of course, the idea.”*. Most employers agreed that the effects of the IPS on SA will be different for people from different departments in the organization. [Employer3]*“I believe there is a difference in SA between the different employees. For example, our staff has much higher absenteeism than Consultancy.*” The difference was often explained by different work stressors.

All employees unacquainted with the strategy expected a decrease in SA. [Employee13] *“I think if you are actively engaged, the absenteeism will go down.”* The awareness about their health situation was also mentioned by the employees as an important factor which could trigger them to cope with their health problems at an early stage. The employees acquainted with the strategy were divided in the expected efficacy of this IPS on SA, some believed it would decrease SA others thought the strategy was without any obligation and dependent on the type of person or work situation.[Employee11] *“So the advantage of doing office work is that you have less chance to get back/knee/joint complaints in comparison to the work outside, which is usually in cramped positions, or heavy lifting ... that is the difference.”* Both employers and employees expected a different effect of this IPS on SA for employees in different departments often dependent on the type of work.

#### IPS effect on future LTSA

All employers acquainted with the strategy had positive expectations about the IPS efficacy on LTSA, however, often the once every three-year frequency was mentioned as inadequate to truly capture LTSA. They also felt that the IPS could not be the only strategy for the prevention of LTSA. [Employer3]*“I am feeling confident about the preventive strategy as part of something larger. I think if you do it alone, well maybe, I do not know, for me it is more than that. Also to make it alive.”* Some employers unacquainted with the strategy mentioned the efficacy of this IPS was also dependent on the cause of SA. The efficacy was questioned by one due to the belief that LTSA was mostly related to cancer or other serious health conditions. Another employer added that LTSA is caused by psychological as well as physical complaints and therefore a more holistic strategy is needed than solely this IPS.

Employees had varying opinions about the possible effects of the IPS on LTSA independent of acquaintance/unacquaintance with the strategy. Some thought it would be effective in the long-term, while others had different reasons to believe that IPS would not be effective in decreasing LTSA. Some employee associated physical complaints primarily with the cause of LTSA and therefore the efficacy of this IPS would be less. [Employee15] *“I think that you select the people who have periods of short-term SA. There may be two or three people who are mentally ill for a long time, but most people who are ill for a long time have physical complaints or health complaints directly.”* In addition, we noticed that some employees, regardless their level of acquaintance with the IPS, were apparently unaware of the OHP consultation being part of the IPS. [Employee10] *“Then it is not just filling in that paper, but then you get the feeling that you are actually being looked at medically.”* The OHP consultation is an essential part of the IPS because it makes sure the employees receive early treatment when they get a high score on the Balansmeter, which means a high risk for future LTSA. This unawareness of the OHP consultation could have affected the views of the employees on the effectiveness of IPS on LTSA.

#### IPS effect on health

Many employers independent of acquaintance/unacquaintance with the strategy believed in a positive effect of the IPS on health through greater awareness about the health situation of employees. Some employers acquainted with the strategy expected a larger effect on psychological health rather than physical health. [Employer4] *“My estimation would be that the impact on psychological symptoms is greater than on the physical symptoms.”* Some employers unacquainted with the strategy were more concerned by the subsequence of the screening questionnaire and the possible overestimation employees could make about their health.

Employees who were acquainted with the IPS were almost all positive about the potential effects on health, but for different reasons. For some employees, their health would benefit through lifestyle advice, for others it was related to an improvement in psychological health. [Employee10] *“And if your diet is good and you feel good and you are physically right, SA will also be less.”* [Employee15] *“That the psychological symptoms will be picked out and you could do something with that, then I think you can really benefit from it.” Some* employees who were unacquainted with the IPS were less positive about the effects on health. Often they focused only on the screening questionnaire and forgot the early consultation. [Employee18] *“Filling in a checklist does not affect my health.”* The consultation with the OHP is considered to be a prerequisite for health improvement but is often not a visible part of the IPS for the employees who are not identified as being at high risk for future LTSA and invited for an early consultation.

### Side effects & barriers

#### Employer views

Almost all acquainted employers were unaware of the costs of this IPS. However, independent of acquaintance/unacquaintance the costs of this IPS were not considered an important factor in the decisions for implementation by many employers. The focus was less on costs and more on the social revenue resulting from being a good employer. [Employer3] *“I think it’s worth the investment from several perspectives. It can of course work preventively, but it is also a bit of awareness, so also good employment practices.”* The employers had different opinions about the Return on Investment (ROI). The ROI of an intervention was not seen as important by everyone; it becomes more important if the costs of an intervention are higher.

Most employers regardless their level of acquaintance with the IPS, expected and often favored a short-term increase in general healthcare use followed by a long-term decrease in healthcare use. They expected more efficient or tailored care for their employees. [Employer2] *“I expect less health care use. At least, more efficient use of care. That people do not wait too long but rather ask for help.”* Few employers unacquainted with the strategy mentioned that this was dependent on the health subject and on the way, employees were confronted with their health status. None of the employers perceived the expected increase in healthcare use in the beginning or stability in healthcare use, before and after implementation as a barrier.

#### Employee views

Almost all employees who were acquainted with the IPS did not perceive a payment for preventive care as something negative; according to them, this care was inevitable. In the Netherlands, care is paid from your obligatory deductible excess, therefore it is expected that people are used to paying the initial part of their healthcare. The employees mentioned that it would be better to receive care at an early stage, to protect their health and prevent higher costs. [Employee16] “*If my health is getting worse, I will have to pay at any given time. It’s for your own health, come on!*” Employees who were unacquainted with IPS had a different perception of the costs of preventive care. The employer was expected to pay for preventive care if it was related to work issues; the employee was expected to pay for health issues not related to work. The deductible for preventive care was definitely not seen as a restraint to use care by most employees acquainted with IPS. They thought their salary was high enough to pay for the care needed. High costs for preventive care were mentioned as a barrier by some of the employees unacquainted with IPS, mainly that their salary would not be high enough to afford preventive care. Almost all employees agreed that it would be positive if the employer would pay for the preventive care but often added that it would never happen if the costs were too high. Few mentioned that they would only agree to this if it was work-related and not self-inflicted. [Employee13] *“If I break my leg on a football field because I’m playing football, then I believe I have to pay for it myself.”* A division is clearly made by the employees if SA is a result of their private actions or work-related.

Some employees acquainted with IPS expected less healthcare due to preventive actions, which could also result in less use of acute healthcare. [Employee16] *“I think less. If someone starts to get sick, sickness can be tackled early.”* Other employees acquainted with IPS mentioned that it would depend on the type of person or how they would interpret the advice from the OHP. The employees unacquainted with IPS all expected more healthcare use. Almost all employees believed that more people would use preventive care if the employer paid for it. Few mentioned that this would not make a difference or that it was dependent on the type of person.

#### Barriers related to adoption

Few employers independent of acquaintance/unacquaintance IPS mentioned possible privacy concerns from employees as an issue and the current privacy debate which could result in employees not wanting to share their personal information. Some employers unacquainted with IPS mentioned the importance of persuading the line managers to stimulate the adoption. By some, the need for positive framing of the strategy was mentioned to support the adoption of this strategy by the employees. [Employer6] *“Currently it has a negative feeling; we need to decrease SA. Turn it around; we have a fun instrument which will benefit your work process, health and create a good work environment”.* Some unacquainted employers mentioned the importance of a validated questionnaire. The employers mentioned different barriers that could inhibit the adoption process of the IPS.

Often employees independent of acquaintance/unacquaintance with IPS mentioned privacy as the biggest barrier to filling out the questionnaire. [Employee15] *“I think there are a lot of people who may be hesitant or afraid that it is not confidential.”* The interviewees had no problems filling out the screening questionnaire but assumed that other people might have, as they may be afraid the information would be made available to the employer. [Employee20] *“So it really has to be a doctor-patient relationship. Fully confidential.”* General privacy breaches were also mentioned as examples that nothing is confidential nowadays. All acquainted employees believed their privacy was protected when they participated in the IPS. A few mentioned being reassured that the physician practices doctor-patient confidentiality and that a university was involved, which also increased the trustworthiness. Some added that there was a possibility the employees would not be completely honest when filling out the screening questionnaire if they were scared of privacy breaches or the employer finding out. The unacquainted employees could not judge the confidentiality of the IPS, because they felt they did not have enough information. However, the protection of personal information was mentioned as highly important.

Almost all employers regardless their level of acquaintance with the IPS, had confidence in the effectiveness of the IPS, but often they added a side note. Some employers acquainted with IPS mentioned that the strategy was only effective if a lot of employees participated or that the strategy needed to be part of something bigger to prevent LTSA. [Employer1] “*In principle a lot, if there are enough people who cooperate with the IPS I think it can have an effect on the absenteeism figures.*” A few employers unacquainted with IPS mentioned they were positive but would like to have more insight into the results of the IPS or phrasing of questions from the screening questionnaire. For one employer who was unacquainted with IPS, the lack of confidence in the effectiveness of the IPS seemed to be prompted by the lack of knowledge about the implementation strategy of the IPS and ignorance about potential effects of the IPS.

Nearly all employees, regardless their level of acquaintance with the IPS, had confidence in the effectiveness of the IPS. Some employees acquainted with IPS mentioned this was due to their own positive experience with it. However, conflicting answers were given while some employees had less confidence in the strategy. Some expected that employees would not be honest about their health situation and others thought they would be honest. [Employee17] “*Why do you need to conceal something? No, it is only to get better. Your health is important.*” According to many employees, the level of confidence in the strategy was determined to a large extent by the honesty of the employees when filling out the screening questionnaire.

Most employees regardless their level of acquaintance with the IPS, did not worry about discrimination or stigmatization related to the implementation of this IPS due to the perception that no personal information from the screening questionnaire would be shared with other employees. [Employee13] “*If it works well nothing. Because nobody will know. I will know and the person who receives the answers from the Balansmeter will know. So if all goes well, that does not have any influence in the workplace.*”. They trusted that their risk score for LTSA would not be shared with colleagues and kept personal, so therefore discrimination would not be possible.

#### Practical barriers related to the implementation

Employers independent of acquaintance/unacquaintance IPS often indicated that finding time to fill out the screening questionnaire was considered a large barrier. Some employers acquainted with the IPS also mentioned the old-school look of the questionnaire and lack of an estimated completion time on the first page. A majority of the acquainted employers mentioned the low-frequency of the questionnaire as an issue. [Employer1] *“It is a nice tool to measure something and then to do something with that measurement, but that measurement is only 1 time in 3 years so yes …*” It only gave them a snapshot of the health state of their employees at one particular time, so they added more general interventions for the whole department. The use of emails as the only correspondence method was seen as a barrier by the unacquainted employers. They mentioned that communication about the IPS in general should be encouraged more.

The employees acquainted with IPS often mentioned they were not able to fill in the screening questionnaire at work due to the time investment. [Employee13] *“When we receive a questionnaire, it is impossible to say am going to stay behind my computer to fill in this questionnaire for the next hour”.* For the acquainted employees, the use of email was mentioned as a large barrier. They often did not have time to check their email regularly. “*Usually I see it via e-mail, when I look at my own situation, when I return after a weekend, sometimes I have 200 e-mails*” [Employee10]*.* Therefore, posters were mentioned as a tool to provide employees with the necessary information about the IPS. They also perceived the frequency of the screening questionnaire as a barrier, while their general state of health varied over time and could not be grasped once every 3 years. Some employees unacquainted with IPS mentioned the importance of company support for this strategy and one mentioned possible fear of discontent when care is only provided to certain individuals A summary of the most important results is provided in Table 3.

**Table 3:** Summary of the overall findings

THEMES	SUB-THEMES	Employers	Employees
VALUES	Perceived responsibility for SA	Large responsibility when related to work-related/psychological complaints	Shared responsibility depends on work-related or non-work-related issues
	Perceived responsibility for health	Shared responsibility depends on work-related or not work-related issues	Shared responsibility depends on work-related or not work-related issues
	Presence of a health culture	Safety culture, focused on physical health	Safety culture, focused on physical health
FACILITATORS	Effect of IPS on future SA	Awareness in employees’ health	SA will decrease
	Effect of IPS on future LTSA	Majority positive but dependent on the type of causes of SA	Mixed feelings, dependent on the type of cause of SA
	Effect of IPS on health	Larger effect on psychological health	Positive about the effects on health
SIDE EFFECTS & BARRIERS	Costs (for this IPS)	Not considered an important factor	N/A
	ROI	Only important if the costs are high	N/A
	Costs and benefit balance	Within normal ranges	N/A
	Healthcare costs	Increase in costs, followed by a decrease	N/A
	Healthcare use	Decrease, due to efficient use of healthcare	Decrease/increase, due to preventive actions
	Costs (own payment for preventive care)	N/A	Often seen as necessary
	Restraint own payment for use of preventive care	N/A	No restraint only if salary is insufficient
	Attitude towards employer if he pays for preventive care	N/A	Positive, some only when the health issue is work-related
ADOPTION BARRIERS	Trust in the privacy of information	Fear of employees not willing to share personal information	Fear of spreading personal information to the employer
	Confidence in the IPS	Confidence, with side notes	Confidence, when employees are honest
	Discrimination/stigmatization	N/A	No fear
PRACTICAL IMPLEMENTATION BARRIERS	Insufficient time to fill out the questionnaire	Large barrier	Large barrier
	Communication issues with the IPS	Issues due to the use of e-mails	Issues due to the use of e-mails
	Low frequency of the IPS	Was perceived an issue	Was perceived an issue

## Discussion

This study aimed to explore expected and perceived facilitators and barriers of an evidence-based indicated prevention strategy (IPS) for the reduction of future LTSA. We focused on employers and employees from large Dutch companies (acquainted and unacquainted with this strategy) and gained insight into the facilitators and barriers employing a qualitative study using semi-structured interviews with 20 employers and employees. The topic list was based on earlier research regarding worksite health preventive interventions and included a broad scope of topics and themes [18–27]. Purposive sampling was used to include respondents at different levels of an organization. Overall, the findings showed that, in general, the employers and employees had positive expectations concerning the effects of this IPS; in particular, the awareness provided by the screening questionnaire about the health situation of employees was appreciated. The ability of a preventive intervention to create awareness about the health situation was also mentioned by Goetzel et al. [30]. All employers and employees saw a potential benefit from the IPS for SA and psychological health. A large barrier according to the employers and employees was the fear of a privacy breach, the fear of spreading information about the health situation of employees to the employer. The explored facilitators and barriers showed to be related to personal or company values.

### Values

The values as studied in the current study may be related to ethical dilemmas due to the dependent/unequal relationship between employer and employees [19]. Values like the perceived responsibility for SA and health may be involved with ethical issues, with the interests of the employer and employee needing to be balanced [31]. To determine whether something is perceived as a facilitator or barrier for this IPS, underlying values in an organization may be of great importance. This is in line with Beer et al. (1990) who stated that the organizational roles and the imposed responsibilities by the organization are very important in explaining the attitudes and beliefs of the individuals [32]. The health culture in an organization is presumed to be of great importance for the adoption of an intervention and perhaps also for its implementation [33]. While the underlying values are associated with the facilitators and barriers, these values could facilitate or hinder the adoption and implementation of the IPS under study. Our themes and sub-themes related to expected facilitators and barriers of this IPS cannot, therefore, be seen as separate items that can be fixed on their own.

We found that employers and employees have in many cases different and opposing perceptions on the responsibility for SA and health. The employers feel primarily responsible when the SA or health impairment is related to work or psychological, and the employees feel responsible when their SA or their health is being affected by their life choices. These results are similar to the findings of Van Berkel [19], which also suggest that employers feel responsible for a healthy working environment and mental health aspects and the employees feel responsible for their lifestyle (and also perceive this as their autonomy). This division sounds logical, however, in reality, there is often no clear boundary between work-related and non-work-related SA or health complaints. It may be difficult to determine whether SA is work-related or not, particularly in the case of psychological or psychosocial health problems (which are multifactorial) [34]. This makes the responsibility for SA and health hazy for both the employers and employees and may hinder the adoption or implementation of preventive strategies.

The perceived responsibility for (types of) SA and health complaints seemed to be influenced by the health culture of an organization, especially for employees. The health culture was often aimed at improving physical health (e.g. eating healthy, enough exercise, safety) and less about psychological health improvement. The employees believed the employer was not responsible for SA or health related to psychological complaints, only safety at work. Possibly the health culture helped frame the way employees view their responsibility for SA/health and the responsibility for the employer. A qualitative study by Tonnon et al. [35] showed similar results regarding the culture in the construction industry. Employees felt discouraged to openly discuss their health complaints and were afraid that other employees would see them as vulnerable. Goetzel et al. [30] explained that when an organization lacks a supportive culture, health improvements that are sponsored by the employer are unlikely to give the desired results. Possibly, due to the lack of a health culture based on physical as well as psychological health the employees do not feel supported with their psychological complaints and will therefore not comply with interventions to improve this or even create a blind spot.

To create support for this IPS and make its adoption and implementation easier, a more holistic health culture is expected to be beneficial. Therefore, the first steps could be to create more awareness of psychological health problems in the organization and encourage employees to disclose such complaints to the OP. Changing the health culture prior to implementation of the IPS seems not realistic. Nevertheless, employers should be informed/stimulated/made aware of the impact of a health culture on employee participation in health interventions such as this IPS.

### Facilitators and barriers

Both employers and employees mentioned they had positive expectations about the effectiveness of this IPS, but the effect on future LTSA was not acknowledged by many employers. Preventing LTSA was perceived as something larger than this IPS, especially due to the low-frequency period of the strategy expected to be insufficient. Moreover, some employers also did not believe this strategy would be effective to prevent LTSA as, according to them, LTSA entails physical as well as psychological problems or solely physical problems, while the prevention of physical problems not being the focus of the IPS. Also some employees also associated LTSA more with physical complaints. Because the IPS was not considered useful for physical complaints, the overall efficacy related to LTSA was expected low. This is in contradiction with the scientific literature, showing that LTSA is most often related to psychological health complaints and would therefore be very appropriate [36, 37]. However, while the employees performed physical demanding tasks this could have resulted in more physical complaints than psychological complaints resulting in LTSA [38]. Nonetheless, there seems to be a strong association with psychological complaints and LTSA which may additionally to physical complaints be relevant for employees performing physically hard work. Therefore, less awareness about the LTSA risk factors might be regarded as a barrier for proper assessment of the impact of this intervention, and as such a barrier hindering implementation.

Another striking observed perception was related to employees’ ideas about the concept behind the IPS. Many employees only mentioned the screening questionnaire and were unaware of the subsequent early consultation with the OHP for high-risk employees, followed by a health intervention, which is essential for the IPS to be effective. Employees often did not know the full content of the IPS, which may have resulted in an underestimation of its potential effectiveness. Even though the employees received all the information about the IPS from their companies, it is understandable that they forgot about steps 2 and 3 because only high-risk employees are invited for those steps and will only experience them when they agree to meet the OHP for an early consultation. Similar results were found in a comparable preventive study, also in an occupational health setting, albeit focusing on a different outcome (Cardio Vascular Disease (CVD)). Here, the employees were aware of the general goal of the intervention, decreasing the risk of a certain outcome, however, they were less informed about the approach of the intervention [35]. This lack of knowledge about the intervention may also be a barrier for implementation.

Often both employees and employers mentioned the fear of a privacy breach as a barrier for this IPS. However, in a similar study conducted amongst a similar group of employees regarding the elevated risk for CVD, no privacy fear was mentioned [35]. Possibly the factors explaining the elevated risk of CVD are often physical and therefore employees are less sensitive about the possibility that this information is leaked to the employer. A study on a digital mental health intervention at the workplace showed that stigmatization of mental illness is still a major problem [39]. As LTSA is often associated with psychological or mental issues, stigmatization and sensitivity of information may play a larger role in this context than in the context of CVD, and this may explain the importance of the fear of a privacy breach as an important barrier for the implementation of the IPS intervention.

Some of our findings can be related to Fassier [21], where he investigated the barriers and facilitators of work disability prevention programs and combined his results in a framework. This framework includes complexity, needs, legislation, resources, organizational practices, professional practices, values, and benefits/risks [21]. A remarkable difference is the needs factor, a facilitator in the framework of Fassier [21], but more or less implicitly present in our findings. According to our results, the need for the employer to implement the IPS is clear, there is a need for the employers to save costs by reducing days of SA and occupational healthcare. The needs for the employees are less apparent, while the employees only receive a high or low risk score from the screening questionnaire, and only after the consultation with the OHP will hear how their scoring has been determined. Clear needs can only be tangible when people are aware of their current health situation. Therefore, the needs regarding this IPS are not yet visible for the employees because of its preventive nature, and employees do not see themselves already as at high risk to become chronically ill [28]. A high-risk score does not give the employees a clear overview about their current health state like e.g. the screening for lung cancer is able to. This is also in line with a coronary risk perception study, which showed that the understanding of the ‘high-risk score’ was mainly based on personal experience and often unrealistic and dichotomous [40]. Attaching meaning to the outcome ‘high-risk’ was perceived to be difficult also according to a Dutch construction worker study [27]. Therefore, the early consultation with the OHP will be crucial to address the meaning of the high-risk score and the associated health/personal issues. This can give the employees more clarity about their health status and their needs regarding the improvement of their health to prevent future LTSA.

### Strengths and limitations

We took several efforts to increase the robustness of the results, including e.g. peer review and attaining saturation of the interview data. To increase rigor and validity a computer program was used to ensure a systematic analysis and audiotapes were used to provide objective recordings. The selection process of employers and employees was based on purposive sampling. The employees were first approached by a colleague from the occupational healthcare service, which they knew and possibly this created a sense of trust between the respondents and the interviewer. It is therefore likely this was fruitful for more accurate and rich information. However, the use of purposive sampling can lead to selective sampling and therefore might not provide an all-encompassing representation of the population. Nonetheless, in our opinion, this research included a wide sample variety in terms of acquaintance with the strategy, male/female employers, varying education levels from the employees, and the fact that saturation was reached. However, since the number of study participants was small (11 employees, 9 employers), we need to be cautious in interpreting these study results.

It is important to note that all employees in our sample were male, which is due to the large proportion of males in the participating organization. This may have influenced the feelings and perceptions about SA, health, and healthcare-seeking behavior. As shown by Thompson et al. [41], men are less likely to seek help for general care compared to women. A similar study with male construction workers also showed that men where less open in addressing their health issues compared to women [35]. Therefore, we need to be cautious in translating these findings to female employees.

Furthermore, studies have shown that a higher education is positively association with preventive behavior and lower education positively with non-seeking treatment [42, 43]. The education level of the respondents was not asked, but based on the job levels, we expect the education level of the employees to vary from low to high and we assume that most employers had high levels of education. As we do not have reliable information about educational level, the relation between education level and values, facilitators and barriers of this IPS could not be determined.

### Transferability of the results

So far, only a few approaches to prevent LTSA, based on the principles of indicated prevention, are described in the literature [14, 15, 17]. As these trials have been conducted either in the Netherlands or in Finland, which has a similar social security system to the Netherlands, we might expect that the explored facilitators and barriers are applicable to a great extent to these or other indicated preventive strategies for SA.

The IPS was implemented in organizations where the occupational service level is high compared to other organizations in the Netherlands. Therefore, we might assume that the trust in the occupational service under study was relatively high. We could hypothesize that for other organizations, with a lower level of occupational service, the aspect of trust in the occupational health service provider or health professionals might be even a greater barrier. Especially with regard to privacy-related issues, which rely strongly on trust, it can be an important barrier for employers and employees when the occupational service is considered to be less trustworthy. Additionally, the respondents were all working for large companies, and therefore it can be expected that some answers will be different for small medium-sized enterprises (SME). In particular, questions related to privacy may be seen differently if employers are less distant from their employees.

Although facilitators and barriers are likely to be intertwined with national legislation, in general, most countries in which the employer is responsible to pay for sick workers (such as northern European countries) have a high perceived incentive to implement a preventive strategy to prevent future LTSA. There is a gradient in employer responsibility for sick pay in European countries in terms of duration and liability (the difference between a few weeks to 2 years). A shift in a low or high incentive to decrease the risk to become chronically ill will depend on the period of sickness benefit. A short period will generate a high incentive for employees and a lower incentive for employers to prevent future SA. Implementing a preventive strategy will then be more important for employees. The applicability of our findings is of limited value for countries where there is almost no social security system, with regards to financial compensation of SA. This is because the responsibility for SA and the costs are shifted solely towards employees, which does not give the employers an incentive to implement a preventive strategy to prevent future LTSA.

### Recommendations for future research

As far as we know, this was the first explorative study, investigating expected facilitators and barriers of an indicated prevention strategy for the prevention of LTSA. We used a semi-structured topic list which might have resulted in more narrow scope of answers. Although the topic list was carefully based on existing literature and other barriers have been consistently asked and explored. Future studies should consider using more open-ended questions to have a broad overview of all possible facilitators and barriers.

The educational level of our respondents was not taken into account in the current study, which may be of value for explaining the different views of respondents among themselves. For future research, it would be interesting to investigate this further. The respondents from the current study all worked in large organizations and therefore it would be interesting for future research to compare the results from this study with SMEs, to assess whether the same facilitators/barriers are visible or others appear.

### Recommendations for practice

This study reveals important issues to improve the future adoption and implementation of this IPS. Educating the employers and employees regarding the true origin of LTSA and the three different steps of this IPS is an important first step towards better adoption and implementation of IPS. The employers and employees may then better understand the way this IPS works and how they could benefit from it and use this strategy to its full potential. Communication is a very important medium to address these apparent perceptions and should not only be done by the use of emails; posters can be a perfect medium for people who do not have time to read emails. Strategic communication, which entails education, motivation, market program offerings, and building trust is seen as one of the most important ways to maintain an effective intervention. The communication needs to be tailored and targeted so employers and employees of different age, sex, and education levels find the intervention appealing [44]. As this IPS focuses on high-risk employees who do not show clear signals of ill-health yet, the needs of these employees are not clearly visible for managers. It is therefore very important for an organization to use strategic communication and explicitly explain the purpose of this strategy to employees and employers alike.

To meet the concerns about privacy, the communication about this IPS needs to be transparent and embedded in the health culture of an organization. This might be a very difficult and long process, therefore it is probably more feasible to start with improving the lines of communication within an organization to provide all the information needed, concerning the privacy issues of employees regarding this IPS. To stimulate adoption, the health culture of an organization is very important and needs to include psychological as well as physical health, since it has an inevitable connection with LTSA [36]. This may provide an opportunity to openly discuss the feelings of responsibility for SA and health which can give more direction to the health needs of employees.

## Conclusion

To conclude, all employers and employees indicated that they were positive about the effects of the IPS regarding SA in general. However, some important barriers were identified, mainly related to privacy issues and different observed discrepancies with regard to the IPS content and the true nature of risk factors associated with LTSA. To further facilitate the adoption and implementation of this IPS, these prevalent and often strong perceptions regarding the nature of LTSA and the content of this IPS need to be addressed, as the effectiveness of the approach will likely be underestimated and the preventive activities misunderstood. The fear of a privacy breach was mentioned as the most important barrier of this preventive strategy and deserves the utmost attention before and during implementation.

## Supplementary Information

**Additional file 1:.** Topic lists used for the semi-structured interviews.

**Additional file 2:.** Overview of the themes and codes from the interviews.

## Data Availability

The datasets generated and/or analyzed during the current study are not publicly available due to the personal information and sensitive information from employers and employees, comprehended in the interviews and which could in theory be might be traced back to individual respondents. The data is only available on site after contact with the corresponding author on reasonable request, to ensure data access complies with the procedures of the General Data Protection Regulation (GDPR).

## Abbreviations

LTSA: Long-term sickness absence
SA: Sickness absence
IPS: Indicated prevention strategy
OP: Occupational Physician
OHP: Occupational Health Professional

## References

[1] Henderson M, Glozier N, Holland EK (2005). Long term sickness absence. BMJ..

[2] Taimela S, Justen S, Aronen P, Sintonen H, Läärä E, Malmivaara A, et al. An occupational health intervention programme for workers at high risk for sickness absence. Cost effectiveness analysis based on a randomised controlled trial. Occup Environ Med. 2008;65(4):242-8. 10.1136/oem.2007.033167 PMC2564864 17933885

[3] van Amelsvoort LGPM, Jansen NWH, Kant I. Addressing long-term sickness absence: moving beyond disease, illness and work-related factors for effective prevention. Scand J Work Environ Health. 2017;43(1):1-4. 10.5271/sjweh.3605 27911453

[4] Rijksoverheid [internet]. The Netherlands; [publisher unknown]; 2020. Hoeveel loon krijg ik doorbetaald als ik ziek ben?; [cited 2019 sept 5]; Available from: https://www.rijksoverheid.nl/onderwerpen/ziekteverzuim-van-het-werk/vraag-en-antwoord/hoeveel-loon-krijg-ik-doorbetaald-als-ik-ziekben.

[5] van Amelsvoort LGPM, Jansen NWH, Kant I (2017). Addressing long-term sickness absence: moving beyond disease, illness and work-related factors for effective prevention. Scand J Work Environ Health.

[6] Vargas-Prada S, Demou E, Lalloo D, Avila-Palencia I, Sanati KA, Sampere M (2016). Effectiveness of very early workplace interventions to reduce sickness absence: a systematic review of the literature and meta-analysis. Scand J Work Environ Health.

[7] van Vilsteren M, van Oostrom SH, de Vet HC, Franche RL, Boot CR, Anema JR. Workplace interventions to prevent work disability in workers on sick leave. Cochrane Database Syst Rev. 2015;10. 10.1002/14651858.CD006955.pub3 PMC9297123 26436959

[8] Vogel N, Schandelmaier S, Zumbrunn T, Ebrahim S, de Boer WE, Busse JW, et al. Return-to-work coordination programmes for improving return to work in workers on sick leave. Cochrane Database Syst Rev. 2017;3. 10.1002/14651858.CD011618.pub2 PMC6464073 28358173

[9] Duijts SF, Kant I, Swaen GM, van den Brandt PA, Zeegers MP (2007). A meta-analysis of observational studies identifies predictors of sickness absence. J Clin Epidemiol.

[10] Wikman A, Marklund S, Alexanderson K (2005). Illness, disease, and sickness absence: an empirical test of differences between concepts of ill health. J Epidemiol Commun H.

[11] Notenbomer A, Roelen C, Groothoff J, van Rhenen W, Bültmann U (2018). Effect of an eHealth intervention to reduce sickness absence frequency among employees with frequent sickness absence: randomized controlled trial. J Med Internet Res.

[12] Lexis MA, Jansen NW, Huibers MJ, van Amelsvoort LG, Berkouwer A, Ton GTA (2011). Prevention of long-term sickness absence and major depression in high-risk employees: a randomised controlled trial. Occup Environ Med.

[13] Division of Public and Behavioral Health [internet]. [place unknown]; [publisher unknown]; 2014. Institute of Medicine (IOM) Classification for prevention; [cited 2019 oct 10]; Available from http://dpbh.nv.gov/uploadedFiles/mhnvgov/content/Meetings/Bidders_Conference/Institute%20of%20Medicine%20Prevention%20Classificationsrev10.20.14.pdf.

[14] Kant I, Jansen NWH, van Amelsvoort LGPM, Swaen GMH, van Leusden R, Berkouwer A (2009). Screening questionnaire Balansmeter proved successful in predicting future long-term sickness absence in office workers. J Clin Epidemiol.

[15] Taimela S, Malmivaara A, Justen S, Laara E, Sintonen H, Tiekso J (2008). The effectiveness of two occupational health intervention programmes in reducing sickness absence among employees at risk. Two randomised controlled trials. Occup Environ Med.

[16] Kant I, Jansen NWH, van Amelsvoort LGPM, van Leusden R, Berkouwer A (2008). Structured early consultation with the occupational physician reduces sickness absence among office workers at high risk for long-term sickness absence: a randomized controlled trial. J Occup Rehabil.

[17] Lexis MAS, Jansen NWH, van Amelsvoort LGPM, Huibers MJH, Berkouwer A, Ton GTA (2012). Prediction of long-term sickness absence among employees with depressive complaints. J Occup Rehabil.

[18] Lexis MA, Jansen NW, Stevens FC, van Amelsvoort LG, Kant I (2010). Experience of health complaints and help seeking behavior in employees screened for depressive complaints and risk of future sickness absence. J Occup Rehabil.

[19] van Berkel J, Meershoek A, Janssens RM, Boot CR, Proper KI, van der Beek AJ (2014). Ethical considerations of worksite health promotion: an exploration of stakeholders’ views. BMC Public Health.

[20] Allender S, Colquhoun D, Kelly P (2006). Competing discourses of workplace health. Health.

[21] Fassier JB. Identifying local obstacles and facilitators of implementation. Handbook of Work Disability 2013(Prevention and Management):441–59.

[22] Envicke B (2012). Investing in a health workforce: the impact of physical wellness on psychological well-being and the critical implications for worker performance. Acad Health Care Manag J.

[23] Gandy WM, Coberley C, Pope JE, Wells A, Rula EY (2014). Comparing the contributions of well-being and disease status to employee productivity. J Occup Environ Med.

[24] Kivimaki M, Head J, Ferrie JE, Shipley MJ, Vahtera J, Marmot MG (2003). Sickness absence as a global measure of health: evidence from mortality in the Whitehall II prospective cohort study. Br Med J.

[25] Hannon PA, Hammerback K, Garson G, Harris JR, Sopher CJ (2012). Stakeholder perspectives on workplace health promotion: a qualitative study of midsized employers in low-wage industries. Am J Health Promot.

[26] Madison KM (2016). The risks of using workplace wellness programs to foster a culture of health. Health Aff.

[27] Damman OC, van der Beek AJ, Timmermans DR (2014). Workers' knowledge and beliefs about cardiometabolic health risk. J Occup Environ Med.

[28] de Brouwer CPM, Verdonk P, van Amelsvoort LGPM, Jansen NWH, Kant I, Widdershoven GAM (2017). Experiences of occupational physicians with the implementation of indicated prevention for long term sickness absence. Work..

[29] Boeije H. Analysis in Qualitative Research: SAGE; 2010. p. 209.

[30] Goetzel RZ, Henke RM, Tabrizi M, Pelletier KR, Loeppke R, Ballard DW (2014). Do workplace health promotion (wellness) programs work?. J Occup Environ Med.

[31] North F, Syme SL, Feeney A, Head J, Shipley MJ, Marmot MG (1993). Explaining socioeconomic differences in sickness absence - the Whitehall-ii study. Br Med J.

[32] Eisenstat R, Spector B, Beer M (1990). Why change programs don’t produce change. Harv Bus Rev.

[33] Randall C, Buys N, Kendall E (2006). Developing an occupational rehabilitation system for workplace stress. Int J Disabil Manag.

[34] Okpaku SO. Essential of Global Mental Health. 2014(ISBN 078–1–10702232-4):138.

[35] Tonnon SC, Proper KI, van der Ploeg HP, Westerman MJ, Sijbesma E, van der Beek AJ (2014). A qualitative study of the anticipated barriers and facilitators to the implementation of a lifestyle intervention in the dutch construction industry. BMC Public Health.

[36] Nielsen MB, Madsen IE, Bultmann U, Christensen U, Diderichsen F, Rugulies R (2011). Predictors of return to work in employees sick-listed with mental health problems: findings from a longitudinal study. Eur J Pub Health.

[37] Dewa CS, Goering P, Lin E, Paterson M (2002). Depression-related short-term disability in an employed population. J Occup Environ Med.

[38] Carroll C, Rick J, Pilgrim H, Cameron J, Hillage J (2010). Workplace involvement improves return to work rates among employees with back pain on long-term sick leave: a systematic review of the effectiveness and cost-effectiveness of interventions. Disabil Rehabil.

[39] Carolan S, de Visser RO (2018). Employees’ perspectives on the facilitators and barriers to engaging with digital mental health interventions in the workplace: qualitative study. JMIR Mental Health.

[40] van Steenkiste B, van der Weijden T, Timmermans D, Vaes J, Stoffers J, Grol R (2004). Patients’ ideas, fears and expectations of their coronary risk: barriers for primary prevention. Patient Educ Couns.

[41] Thompson AE, Anisimowicz Y, Miedema B, Hogg W, Wodchis WP, Aubrey-Bassler K (2016). The influence of gender and other patient characteristics on health care-seeking behaviour: a QUALICOPC study. BMC Fam Pract.

[42] Bristow K, Patten S (2002). Treatment-seeking rates and associated mediating factors among individuals with depression. Can J Psychiatr.

[43] Gulliver A, Griffiths KM, Christensen H (2010). Perceived barriers and facilitators to mental health help-seeking in young people: a systematic review. BMC Psychiatry.

[44] Kent K, Goetzel RZ, Roemer EC, Prasad A, Freundlich N (2016). Promoting healthy workplaces by building cultures of health and applying strategic communications. J Occup Environ Med.

