# Correction to: Exploring expected and perceived facilitators and barriers of an indicated prevention strategy to prevent future longterm sickness absence; a qualitative study among employers and employees

**DOI:** 10.1186/s12889-021-10434-3

**Published:** 2021-02-18

**Authors:** Sophie H. Klasen, Ludovic G. P. M. van Amelsvoort, Inge Houkes, Nicole W. H. Jansen, IJmert Kant

**Affiliations:** 1 Department of Epidemiology, Faculty of Health, Medicine and Life Sciences, CAPHRI School for Public Health and Primary Care, Maastricht University, P.O. Box 616, 6200, MD Maastricht, The Netherlands; 2 Department of Social Medicine, Faculty of Health, Medicine and Life Sciences, CAPHRI School for Public Health and Primary Care, Maastricht University, Maastricht, The Netherlands

**Correction to: BMC Public Health (2021) 21:289**

**https://doi.org/10.1186/s12889-021-10322-w**

It was highlighted that in the original article [1] the numbers assigned to each image depicted in Fig. 1 were miss-numbered in the published version as 2-3-1 instead of 1-2-3. This Correction article shows the correct Fig. 1. The original article has been updated.

**Fig. 1:**
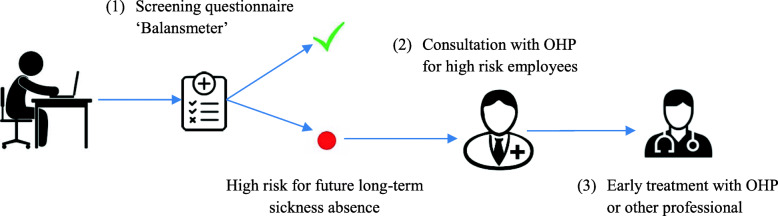
Indicated prevention strategy; prediction and early consultation to prevent future LTSA
