# Supplementary material for: Exploring expected and perceived facilitators and barriers of an indicated prevention strategy to prevent future long-term sickness absence; a qualitative study among employers and employees
Source: BMC Public Health. 2021 Feb 4;21:289. doi: 10.1186/s12889-021-10322-w (PMC7863522; doi:10.1186/s12889-021-10322-w)
Supplement: Supplementary file 1 — Additional file 1:. Topic lists used for the semi-structured interviews. [file 12889_2021_10322_MOESM1_ESM.docx]

# Additional file 1: Topic lists used for the semi-structured interviews

## Interview topics for employers

- Responsibility
  - Responsibility for the health of the employee
    - To what extent do you feel responsible for the health of the employee?
  - Responsibility for sickness absence of the employee
    - To what extent do you feel responsible for the sickness absence of the employee?
    - To what extent do you, as an employer, think you can influence the sickness absence rate of your employees?
- Benefits of preventive strategy
  - Sickness absence
    - What effect do you expect from this preventive strategy on the sickness absence of employees in your company?
    - (If applicable) What effect have you seen from the preventive strategy on the sickness absence of employees in your company?
    - (If applicable) Was this effect different from what was expected?
    - Could the effect on sickness absence be different for different groups of employees?
  - Health
    - What is the meaning of the preventive strategy for different facets of employees’ health?
  - Healthcare use
    - Do you expect more or less healthcare use?
    - Do you perceive more or less use of healthcare as an obstacle for the preventive strategy?
    - How and to what extent did you insure the risk for sickness absence of your employees?
- Culture
  - Does health play a role in the culture of the company?
  - How would you describe the health culture of the company?
- Barriers
  - Trust the method
    - To what extent do you have confidence in the preventive strategy?
    - What do you think about the use of a preventive strategy to prevent long-term sickness absence?
    - Do feedback moments of the results from the preventive strategy contribute to an increase in trust in the preventive strategy?
  - Barriers for the employees
    - Do you expect barriers for your employees to participate in this preventive strategy?
  - Costs
    - To what extent do you have insight into the costs of the preventive strategy?
    - How do you perceive the cost-benefit balance of this preventive strategy?
    - Do you perceive high costs if the benefits are also high?
    - To what extent does the Return On Investment (ROI) play a role in the implementation of the preventive strategy?
    - If the financial benefits are for another person, how do you perceive the costs of this preventive strategy?
  - Other
    - Are there other barriers that can prevent the use of this preventive strategy?

## Interview topics for employees

- Responsibility
  - Responsibility for the health of the employee
    - To what extent is the employer responsible for the health of the employee?
  - Responsibility for sickness absence of the employee
    - To what extent does the employer feel responsible for the sickness absence of employees?
    - To what extent can the employer influence the sickness absence of an employee?
- Benefits of preventive strategy
  - Sickness absence
    - What effect do you expect from this preventive strategy on the sickness absence of employees in your company?
    - Could the effect on sickness absence be different for different groups of employees?
  - Health
    - What is the meaning of the preventive strategy for different facets of employees’ health?
  - Healthcare use
    - Do you expect more or less healthcare use?
- Culture
  - Does health play a role in the culture of the company?
  - How would you describe the health culture of the company?
- Barriers
  - Trust in the method
    - To what extent do you have confidence in the preventive strategy?
    - What do you think about the use of a preventive strategy to prevent long-term sickness absence?
    - Do feedback moments of the results from the preventive strategy contribute to an increase in trust in the preventive strategy?
  - Barriers for the employees
    - Do you expect employees to face barriers to participate in this preventive strategy?
  - Privacy
    - What do you think about the privacy of the sensitive information that is requested in the questionnaire?
    - In your opinion, is the privacy of the sensitive information sufficiently guaranteed?
  - Discrimination
    - To what extent do you believe that the use of the preventive strategy can lead to discrimination/stigmatization?
  - Costs
    - What do you think about paying for preventive care yourself?
    - To what extent would your deductible prevent you from using preventive care?
    - What will the employee think about the costs for preventive care for the employer?
    - Are the costs for the employer an obstacle or an incentive to use preventive care?
  - Other
    - Are there other barriers that can prevent the use of this preventive strategy?
