# Supplementary material for: Exploring expected and perceived facilitators and barriers of an indicated prevention strategy to prevent future long-term sickness absence; a qualitative study among employers and employees
Source: BMC Public Health. 2021 Feb 4;21:289. doi: 10.1186/s12889-021-10322-w (PMC7863522; doi:10.1186/s12889-021-10322-w)
Supplement: Supplementary file 2 — Additional file 2:. Overview of the themes and codes from the interviews. [file 12889_2021_10322_MOESM2_ESM.docx]

# Additional file 2: Overview of the themes and codes from the interviews

- Values
- responsibility of employer for health of employee
- responsibility for health
- safety culture
- culture of change
- culture of fear
- responsibility of employee for health of employee
- responsibility for safety
- responsibility for sickness absence
- Facilitators
- prevention
- personal attention
- coping with sickness absence
- interventions are different for sub-groups
- general effect of preventive strategy
- modified work
- effect of IPS on health
- effect of preventive strategy for different sub-groups
- benefit of a preventive strategy
- stress related to work
- next steps following preventive strategy
- affecting behavior of employees
- need for valid measurements
- long-term sickness absence
- awareness given by the preventive strategy
- effect of IPS on sickness absence
- being a good employer
- office and factory difference
- different types of sickness absence
- Side effects & barriers
- private business
- uncertainty about preventive strategy
- no trust in the manager
- incentive or impediment if employer pays healthcare costs
- barriers strategy
- barriers for employees
- more or less healthcare use as a result of this IPS
- deductible for preventive care
- financial benefits for someone else
- consequences of financial choices
- high costs and benefits
- costs of preventive strategy
- costs and benefit balance
- costs after sickness absence
- medicalization
- need for correct positioning of the preventive strategy
- privacy of sensitive information
- ROI
- discrimination or stigmatization by the use of this IPS
- trust in this IPS
- trust in the safety of sensitive information for employers and employees
- level of insurance for sickness absence
- withhold preventive care if deductible is needed
- employer pays preventive care
- healthcare use as a result of this IPS
- healthcare use differs by age
